# Supplementary material for: Ambient‐Pressure Superconductivity Onset at 10 K and Robust T c under High Pressure in TiNbTaN3 Medium‐Entropy Nitride
Source: Adv Sci (Weinh). 2025 Jun 29;12(32):e06089. doi: 10.1002/advs.202506089 (PMC12407338; doi:10.1002/advs.202506089)
Supplement: Supplementary file 1 — Supporting Information [file ADVS-12-e06089-s001.docx]

Supporting Information

Ambient-pressure superconductivity onset at 10 K and robust *T*_c_ under high pressure in TiNbTaN_3_ medium-entropy nitride

Lingyong Zeng,* Jie Wang, Hongyu Liu, Longfu Li, Jinjun Qin, Yucheng Li, Rui Chen, Jing Song,* Yusheng Hou,* Huixia Luo*

L. Zeng, L. Li, J. Qin, Y. Li, R. Chen, H. Luo

School of Materials Science and Engineering, State Key Laboratory of Optoelectronic Materials and Technologies, Key Lab of Polymer Composite & Functional Materials, Guangdong Provincial Key Laboratory of Magnetoelectric Physics and Devices, Sun Yat-sen University, Guangzhou 510275, China
E-mail: luohx7@mail.sysu.edu.cn (H. Luo)

L. Zeng
Device Physics of Complex Materials, Zernike Institute for Advanced Materials, University of Groningen, 9747 AG Groningen, The Netherlands

E-mail: l.zeng@rug.nl (L. Zeng)

J. Wang, Y. Hou

Guangdong Provincial Key Laboratory of Magnetoelectric Physics and Devices, School of Physics, Sun Yat-sen University, Guangzhou 510275, China

E-mail: houysh@mail.sysu.edu.cn (Y. Hou)

H. Liu, J. Song

*Beijing National Laboratory for Condensed Matter Physics, Institute of Physics,*

Chinese Academy of Sciences, Beijing 100190, China

E-mail: jingsong@iphy.ac.cn (J. Song)


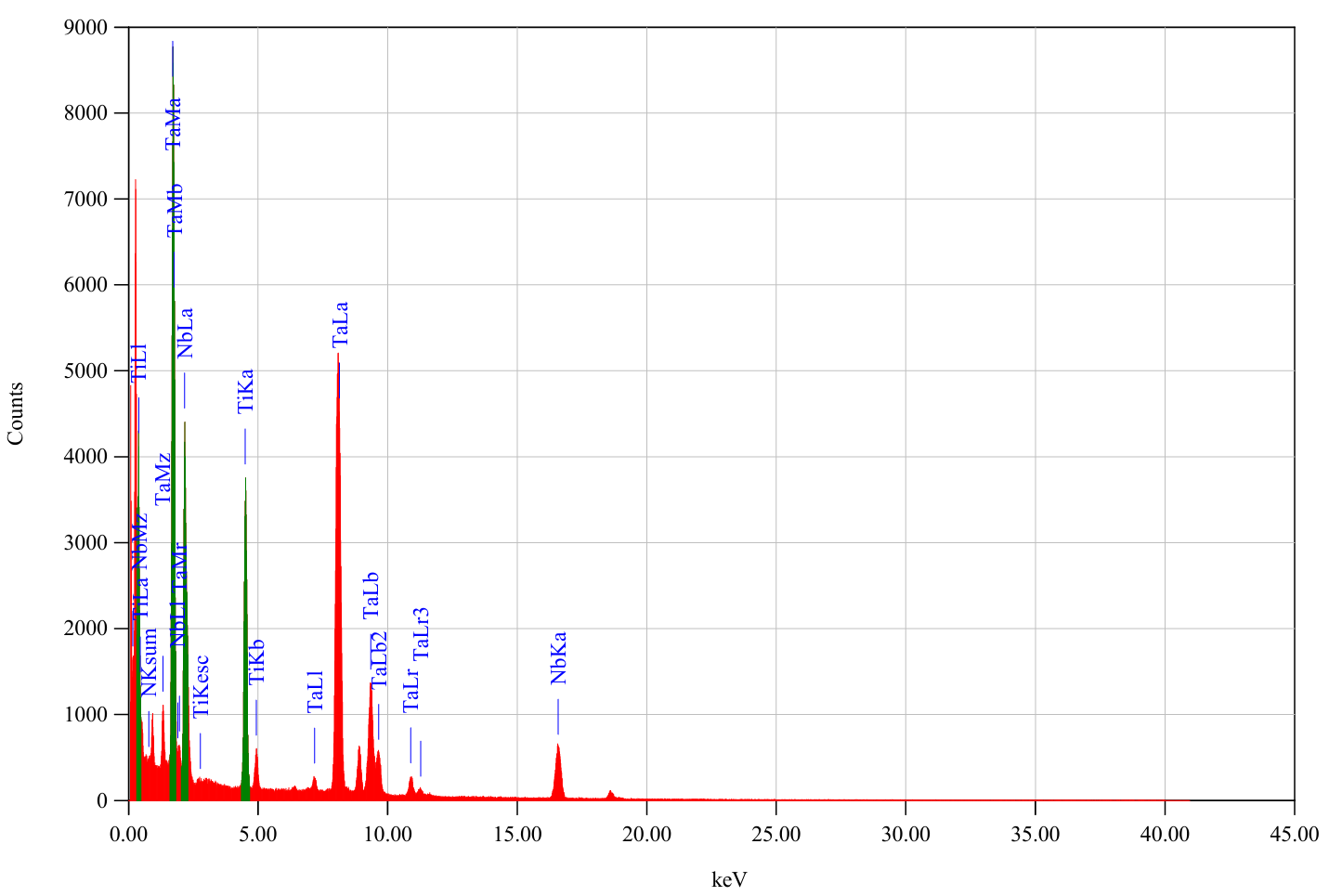


**Figure S1.** EDS spectrum of TiNbTaN_3_ MEN.


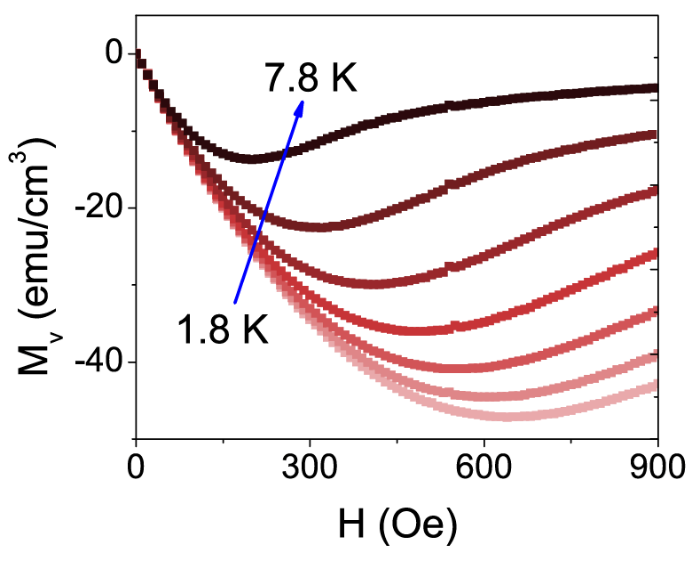


**Figure S2.** Isothermal magnetization curves (M vs. H) in the regime (0 - 900 Oe; T = 1.8 - 7.8 K).


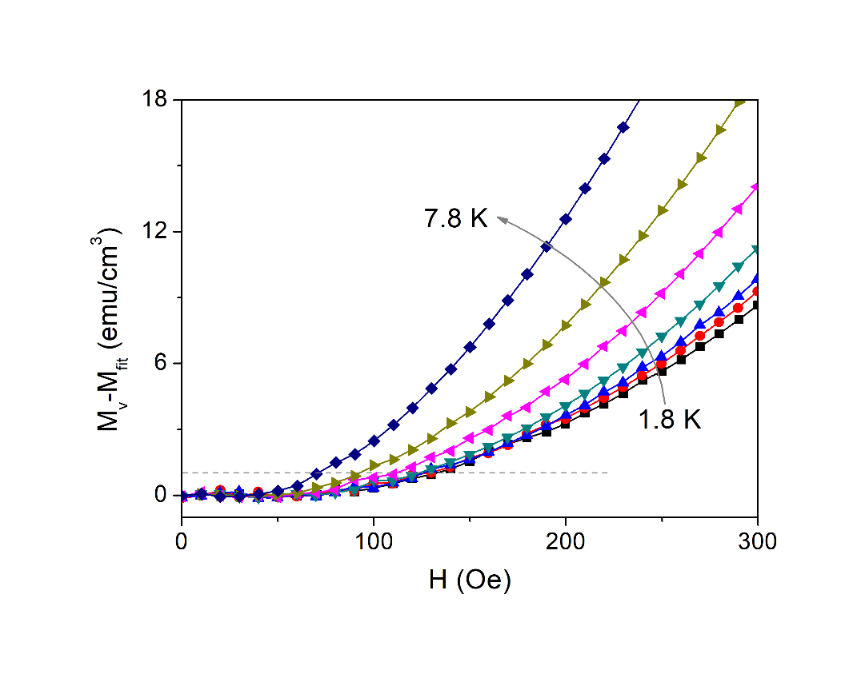


**Figure S3.** The difference between M_v_ and M_fit_ at 0 - 300 Oe under several temperatures for TiNbTaN_3_ MEN.


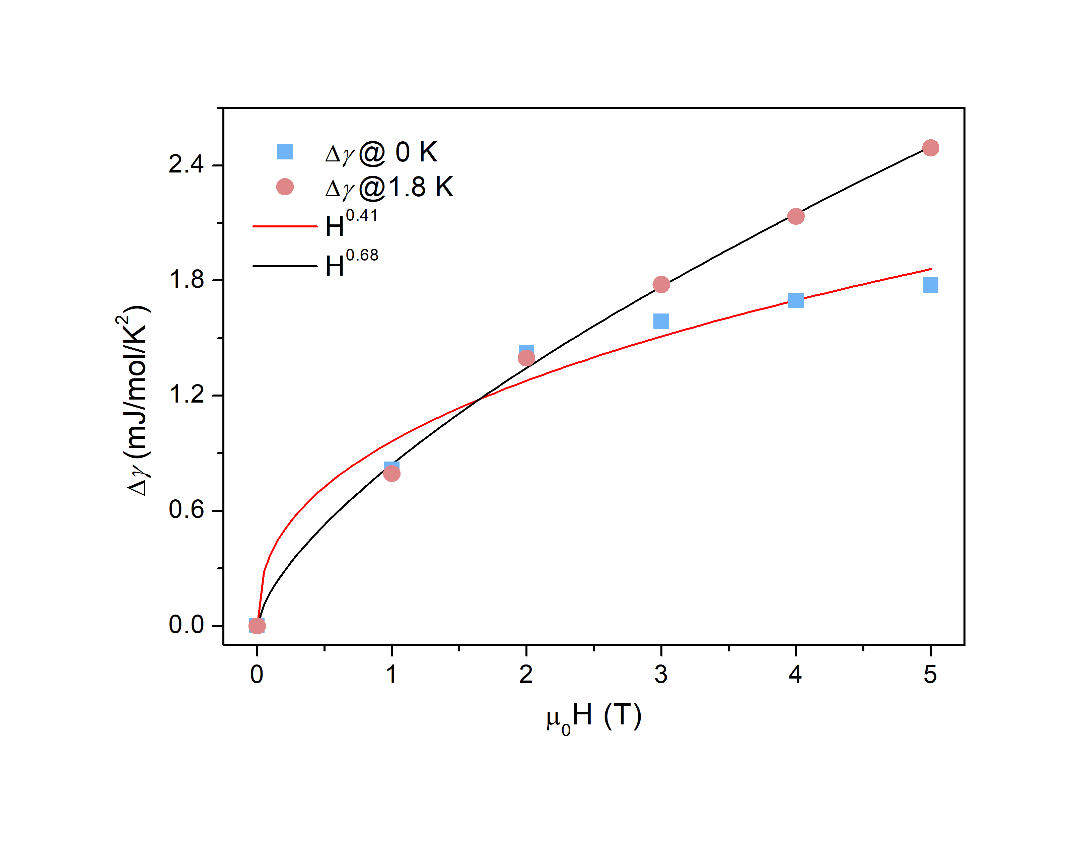


**Figure S4.** The field dependence of the reduced electronic specific heat coefficient ∆*γ* at 0 K and 1.8 K.

**Table S1.** The optimized lattice constants and internal coordinates of TiNbTaN_3_ under different pressures.

| Pressure (GPa) | 0 | 26.8 | 54.5 |
| --- | --- | --- | --- |
| Lattice constants ($\mathbf{Å}$) | 4.3266(3) (*exp.*) | 4.2341 | 4.1596 |
| Internal coordinates | Ti/Nb/Ta (0, 0, 0)  N (0.5, 0.5 ,0.5) | Ti/Nb/Ta (0, 0, 0)  N (0.5, 0.5 ,0.5) | Ti/Nb/Ta (0, 0, 0)  N (0.5, 0.5 ,0.5) |

The lattice constant used at 0 GPa is based on the experimental value. The internal coordinates remain unchanged after structural relaxations under different pressures due to the high symmetry of the crystal structure of TiNbTaN_3_.





**Figure S5.** DFT calculated band structures and DOS (with and without SOC) of TiNbTaN_3_ under different pressures using the cut-off energies of a) 500 eV, b) 550 eV, c) 600 eV.
